# Trophoblast lineage specification in the mammalian preimplantation embryo

**DOI:** 10.1002/rmb2.12333

**Published:** 2020-07-02

**Authors:** Yayoi Toyooka

**Affiliations:** ^1^ Center for iPS Cell Research and Application (CiRA) Kyoto University Kyoto Japan

**Keywords:** Cdx2, cell differentiation, plasticity, trophectoderm (TE), trophoblast

## Abstract

**Background:**

The establishment of the trophectoderm (TE) and the inner cell mass (ICM) is the first cell lineage segregation that occurs in mammalian preimplantation development. TE will contribute to the placenta while ICM cells give rise to the epiblast (EPI) and primitive endoderm (PrE). There are two historical models for TE/ICM segregation: the positional (inside‐outside) model and the polarity model, but both models alone cannot explain the mechanism of TE/ICM segregation.

**Methods:**

This article discusses a current possible model based on recent studies including the finding through live‐cell imaging of the expression patterns of caudal type homeobox 2 (Cdx2), a key transcription factor of TE differentiation in the mouse embryo.

**Results:**

It was observed that a part of outer Cdx2‐expressing blastomeres was internalized at the around 20‐ to 30‐cell stage, downregulates Cdx2, ceases TE differentiation, and participates in ICM lineages.

**Conclusion:**

The early blastomere, which starts differentiation toward the TE cell fate, still has plasticity and can change its fate. Differentiation potency of all blastomeres until approximately the 32‐cell stage is presumably not irreversibly restricted even if they show heterogeneity in their epigenetic modifications or gene expression patterns.

## Graphical abstract

Early blastomere which starts differentiation toward the TE cell fate still have has plasticity and can change its fate to ICM lineages depending on their positional changes.

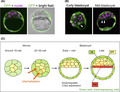

## 1 INTRODUCTION

One of the most characteristic features of preimplantation mammalian embryogenesis is self‐organization; cell fates and their positions are determined not by the distribution of maternal factors in the oocyte, such as the case with flies or flogs, but by the interaction of cells that comprise the embryo. The establishment of the trophectoderm (TE) and the inner cell mass (ICM) is the first cell lineage segregation that occurs during mammalian preimplantation development. TE is an epithelium‐like structure that is formed by trophoblast cells, as it appears on the surface of the embryo to contribute to the placenta, while ICM cells, which arise from the cell clump that is surrounded by the TE layer, give rise to the epiblast (EPI) and primitive endoderm (PrE) approximately one day after the appearance of the TE. For lineage segregation of TE/ICM, mouse preimplantation embryo is known to hold great plasticity at even the 32‐cell stage, while several reports suggest heterogeneity among 2‐ or 4‐cell blastomeres, which subsequently affect cell fate decisions.^1^, ^2^ Traditionally, the positional (inside‐outside) and polarity models have been proposed for TE/ICM specification. In terms of the positional model, the cell position within the embryo (inner or outer) determines cell fate, while the polarity model proposes that the TE cell fate is determined by inherited factors located on the surfaces of the polarized embryo and that are unevenly divided during asymmetric cell division. Both models alone cannot explain the mechanism of TE/ICM specification. Thus, certain researchers have suggested and proposed models that combine these traditional models.^3^, ^4^ Nevertheless, for a population of blastomeres, their behavior is yet to be explained by these hypotheses. In this article, I introduce an overview of mouse preimplantation development referring to the plasticity and heterogeneity of blastomeres, two traditional models for TE cell specification, and a possible model incorporating the latest knowledge. In addition, characteristics and differences of trophoblast stem cells (TSCs) that are derived from early mouse and human embryos are mentioned, which are expected to be useful for research on TE development and its function during the peri‐implantation period.

## 2 AN OVERVIEW OF MAMMALIAN PREIMPLANTATION DEVELOPMENT

### 2.1 Compaction

**FIGURE 1:**
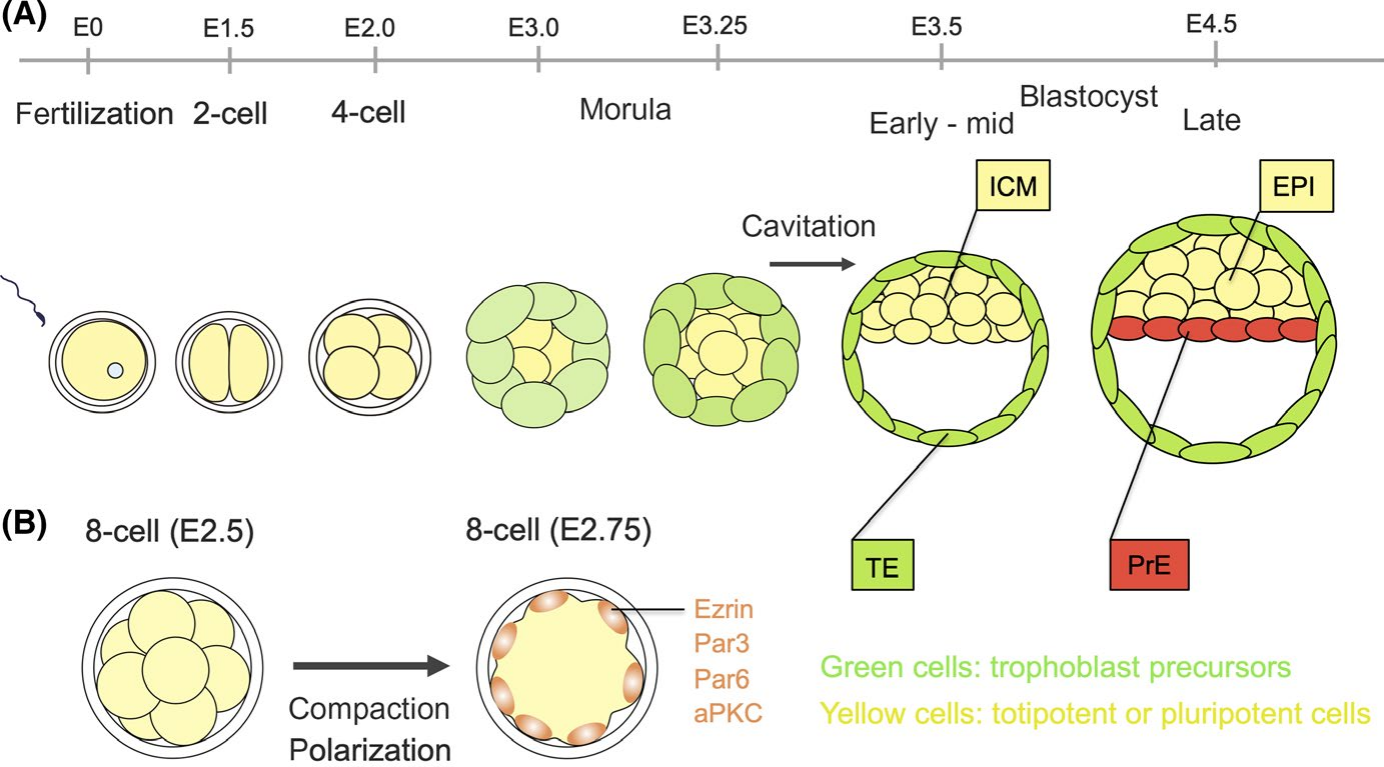
A schematic representation of preimplantation mouse development. (A) The sequence of events during preimplantation mouse development. Morphological change and differentiation of three cell lineages, trophectoderm (TE), epiblast (EPI), and primitive endoderm (PrE) occur before implantation. At around E (embryonic days) 3.5, blastocyst formation occurs, and TE and inner cell mass (ICM) are produced at outside and inside positions of the embryo, respectively. Then, ICM gives rise to EPI and PrE at around E4.5. (B) Compaction and polarization, two major morphological changes, occur at 8‐cell stage concomitantly. Microvilli, ezrin, and polarity proteins such as Par3, Par6, and aPKC are restricted to the contact‐free apical domain of blastomeres. Green cells: trophoblast precursors, yellow cells: totipotent or pluripotent cells.

During mouse preimplantation development, a fertilized egg undergoes cleavage and increases its cell number. At the 8‐cell stage, embryos undergo compaction—the first major morphogenetic event in which the cell‐cell contact area is at its maximum, the cell shape flattens, and the embryo forms a tightly packed cell mass with a spherical outer surface (Figure 1A,B). The most responsible molecule for the compaction and formation of adherens junctions is the cell adhesion molecule E‐cadherin. The addition of neutralizing antibodies for E‐cadherin or the removal of Ca^2+^ ions from the embryo incubation media, which prevents E‐cadherin hemophilic binding, ablates compaction.^5^, ^6^, ^7^ The removal of both maternal and zygotic E‐cadherin with certainty leads to compaction prevention,^8^ while embryos in which only zygotic alleles of E‐cadherin were removed could compact normally due to carryover of maternal E‐cadherin.^9^, ^10^ Further, the removal of maternal E‐cadherin results in delayed initiation of compaction until the late morula stage.^11^ E‐cadherin protein is distributed in the plasma membrane of 8‐cell stage blastomeres prior to compaction and then becomes restricted to the basolateral cell‐cell contact sites during compaction.^5^ Phosphorylation of E‐cadherin has been demonstrated in early mouse embryos to come simultaneously with compaction.^12^ Further, it was found that forced activation of Ca^2+^ phospholipid‐dependent protein kinase C (PKC) by treating 4‐cell embryos with chemicals leads to premature compaction via an E‐cadherin‐based pathway.^13^ These findings suggest that E‐cadherin protein is present prior to the 8‐cell stage and is regulated in a post‐translational manner during compaction. Fierro‐Gonzalez et al showed, using a sophisticated live‐imaging technique, that filopodia is exerted from the apical border of 8‐cell blastomeres and adheres to the top of their neighboring blastomeres via E‐cadherin, and laser ablation of filopodia results in rounding of blastomeres and separation from neighbors.^14^ These observations suggest that filopodia are necessary to maintain cell shape and cell‐cell contact during compaction. Another report showed that pulsatile actomyosin contractility promotes cortical tension that is required for compaction.^15^ Interactions that exist between filopodia that contain E‐cadherin and actomyosin contractility are not yet resolved.

### 2.2 Polarization

Concurrent with compaction, polarization occurs at the 8‐cell stage. Each blastomere of an 8‐cell embryo acquires apical‐basal polarity, which is accompanied by the apical localization of microvilli ^16^ and several molecules, such as atypical protein kinase C (aPKC), PAR3,^17^ PAR6,^18^ and ezrin.^19^ PAR3, PAR6, and aPKC collectively form a complex (PAR3‐PAR6‐aPKC complex), which is known to be essential for the establishment of cell polarity in many cell types (Figure 1B). The most visible alteration during polarization is the restriction of microvilli to the embryo surface; microvilli are localized uniformly at the 8‐cell blastomere before compaction, are excluded from the cell‐cell contact regions, and are restricted to the contact‐free surface of the blastomeres.^16^ Ezrin is an actin‐binding protein and is likewise known to be restricted to the contact‐free surface of the blastomeres during polarization.^19^ Phosphorylation of ezrin is suggested to lead to the restriction of its distribution and the regulation of the formation and stabilization of microvilli. The phosphorylation of the amino acid residue threonine‐567 (T567) of ezrin and substitution for alanine is assumed to be a prerequisite to restrict its distribution, since substitution of T567 for alanine results in the accumulation of ezrin to cell‐cell contact regions, mislocalization of microvilli at basolateral regions, and failure of appropriate formation of cell‐cell contacts that are mediated by E‐cadherin and compaction.^20^ The PAR3‐PAR6‐aPKC complex is also restricted to the contact‐free surface of the blastomeres during polarization. Phospholipase C‐mediated PIP_2_ hydrolysis was also demonstrated to induce accumulation of actomyosin at the apical cortex of the blastomere via activation of PKC, and consequently RhoA. This is a prerequisite for the second phase, in which the PAR3‐PAR6‐aPKC complex accumulates to the apical domain, which excludes actomyosin and forms a mature apical domain.^21^

### 2.3 TE/ICM segregation

At approximately the 32‐cell stage, the segregation of the following first two cell lineages occurs: the trophectoderm (TE) and the inner cell mass (ICM), which appear during the blastocyst stage at outside and inside positions of the embryo, respectively (Figure 1A). TE will generate the future placenta, while ICM gives rise to the epiblast (EPI) and primitive endoderm (PrE), approximately one day after the appearance of the TE. EPI cells are the pluripotent cell population and give rise to the embryo proper, whereas the PrE cells generate extraembryonic tissues. A detailed discussion on the mechanism and models for segregation of TE and ICM cell lineages is provided later in this review.

### 2.4 Cavitation

Cavitation is the second major morphogenetic event that occurs in concurrence with the formation of an outer‐inner configuration. Moreover, multiple small cavities have been found to appear in the intercellular spaces of the embryo^22^ due to exocytosis of vesicles or vacuoles from the basal membrane of the outer cells.^23^ Sodium ions are transported in the inside of the outer layer via transmembrane pumps, and the osmic gradient that is caused by the concentration difference of the sodium ion generates fluid from outside to inside the embryo ^24^, ^25^ and leads to the expansion of small cavities. Interestingly, small cavities only appear between trophoblast cells during cavity formation, and ICM cells are packed in trophoblast cells and appear to never be exposed to fluid in the small cavities.^26^ Small cavities finally fuse into a single cavity, continue to expand, and are thought to supply a necessary force for embryos to hatch from the zona pellucida (ZP). As the blastocyst cavity expands, ICM cells form a single‐cell mass that is located at the embryonic pole. ICM was observed to be covered by thin protrusions that are projected from the outer neighboring TE cells, which seem to cover and pack the ICM during the early to mid‐blastocyst stages. Fleming et al observed the morphology of the early to mid‐blastocyst stages by transmission electron microscopy and showed that the inner cells were covered with processes that were protruded from outer cells, which were presumed to be TE precursors.^27^ Since several studies show ICM that is isolated from the early blastocyst regenerates a TE layer on its surface and that ICM from the late blastocyst forms a PrE layer,^27^, ^28^, ^29^, ^30^, ^31^, ^32^, ^33^ we suppose that these coverings may work as a barrier to protect ICM cells from the extracellular microenvironment and to maintain a pluripotent population, as speculated by Fleming et al.^27^ The protrusions from the TE cells became thin and retracted from the surface of the ICM, and thus seem to be replaced by the PrE layer at the late blastocyst stage.^26^

### 2.5 EPI/PrE segregation

Following TE/ICM formation, the second cell lineage segregation occurs, as ICM cells differentiate into the epiblast (EPI) and primitive endoderm (PrE, Figure 1A). EPI is a cell lineage that maintains pluripotency and gives rise to embryo proper as developmental processes proceed, while PrE mainly contributes to extraembryonic membranes, such as the amnion. PrE precursor appears in a “salt‐and‐pepper pattern” with apparently random distributions in the ICM, and then cell arrangement occurs by cell sorting.^34^, ^35^ PrE cells migrate to the surface of the ICM that faces the blastocyst cavity, and the EPI is enclosed by the TE and PrE.^34^ Segregation of the EPI and PrE is thought to be determined by reciprocal expression of the two transcription factors Nanog and Gata6.^34^ ICM cells first uniformly express molecules that are involved in pluripotency, such as Nanog, Oct4, Sox2, and Fgf4. Then, Fgf4 is secreted from ICM cells in an autocrine manner.^34^ Father, its receptor Fgfr2 and components of its downstream pathway, termed the ERK pathway, are known to be indispensable for differentiation of PrE—based on analyzing knockout mice of these molecules.^34^, ^35^ Gata6 is also shown to be an essential factor for PrE lineage development based on analysis of knockout mice.^36^ In fact, Gata6‐null mice die at embryonic day (E) 5.5‐E7.5 because of defects in the formation of the visceral endoderm, which is a derivative of PrE.^36^ Forced expression of Gata6 induces PrE cell differentiation of mouse embryonic stem (ES) cells, which suggests that Gata6 is a main trigger for the induction of PrE.^37^ In PrE precursors, the binding of Fgf4 to Fgfr2, which is also expressed in ICM cells, leads to activation of the ERK signaling pathway. Increased input to the ERK pathway results in the repression of Nanog expression levels and promotes upregulation of Gata6, and lastly leads differentiation into PrE. EPI precursors continue to express Nanog and secrete FGF4, and are thought to promote PrE differentiation of neighboring cells. However, to complete this story, certain upstream factors that enhance the input intensity from the Fgf4‐Fgfr2‐ERK cascade in the PrE precursors are needed. As with Fgfr2, Fgfr1 was also reported to be utilized for differential transduction of the Fgf4 signal in ICM cells,^38^, ^39^ but as of now, no researcher has determined the mechanisms for how the first difference between the EPI and PrE precursors generates and for how it leads to different input from the Fgf4‐Fgfr2‐ERK‐Nanog axis among each ICM cell. Also, it is still unrevealed how the ratio of EPI: PrE is adjusted in an appropriate range. Further research is required to resolve the entire mechanism for the segregation of EPI/PrE.

## 3 DEVELOPMENTAL PLASTICITY OF BLASTOMERES IN THE EARLY PREIMPLANTATION EMBRYO

Early preimplantation embryo possesses high plasticity, that is, developmental flexibility, tolerate isolation and removal, and rearrangement of blastomeres. For mice, a blastomere of a 2‐cell stage embryo is totipotent and results in a viable mouse.^40^, ^41^ A blastomere from a 4‐cell and 8‐cell embryo cannot develop solely, but can contribute to all tissues when they are combined with other blastomeres as chimera.^42^, ^43^ Blastomeres of embryos at the 16‐ to 32‐cell stage have been shown to change their fates when their positions within the embryo are experimentally changed.^44^ It has been also reported that even individual blastomeres at the 16‐cell embryo stage could differentiate into both trophoblast and ICM and finally generate adult mice when enclosed by tetraploid cells, which suggests that at least some cells at the 16‐cell embryo stage still possess full developmental potency.^45^ Such a developmental flexibility of mammalian preimplantation embryos was also demonstrated using other animals. For instance, single blastomeres of 2‐, 4‐, and 8‐cell rabbit embryos were found to be able to generate live animals,^46^ and single blastomeres of 4‐ and 8‐cell sheep embryos can develop into viable individuals.^47^ In caw, individual blastomeres of a 4‐cell embryo resulted in four genetically identified animals.^48^ These results suggest that cells in mammalian preimplantation embryos similarly retain great plasticity up to approximately the 8‐ and 16‐cell stage.

## 4 HETEROGENEITY AMONG BLASTOMERES IN THE EARLY EMBRYO

Although no critical differences were proposed between blastomeres in mouse embryos in terms of differentiation ability prior to trophoblast/ICM segregation (ie, approximately the 16‐cell stage)—since their full developmental potential and extraembryonic parts were experimentally confirmed, as previously described—several lineage‐tracing studies reveal that some blastomeres in the 2‐ and 4‐cell embryos were biased toward certain cell lineages and did not contribute equally to all cell lineages.^49^, ^50^, ^51^ Further, it was revealed that the status of epigenetic modification was not identical between each blastomere in 4‐cell embryos. Levels of methylation of arginines 17 and 26 of histone H3 (H3R17 and H3R26) differed among blastomeres in 4‐cell stage embryos.^2^ Expression levels of chromatin modifiers Carm1^2^ and PRDM14^1^ were varied among 4‐cell blastomeres, and the injection of mRNA for Carm1 into 4‐cell blastomeres promoted H3R26 methylation and higher expression levels of Nanog and Sox2, and led blastomeres to be positioned into the ICM.^2^ Overexpression of PRDM14 in 4‐cell blastomeres also increased H3R26 methylation and directed blastomeres toward ICM.^1^ These results show that the blastomeres that demonstrate higher methylation levels at H3R17 and H3R26 tend to contribute to the ICM of the embryo,^1^, ^2^ which suggests that the heterogeneity of epigenetic modifications in 4‐cell blastomeres correlate with cell fate and potency. The causes of such heterogeneity in the aspects of cell fate or epigenetic status among 2‐ or 4‐cell blastomeres are not yet revealed. Traditionally, some research groups proposed the “pre‐patterning hypothesis,” in which the early blastomere inherits some factors that are unevenly distributed in the fertilized egg, and the number of inherited factors determine the cell fate of the blastomere‐like “maternal factors” in eggs of vertebrates. However, this hypothesis did not establish consensus among researchers of the preimplantation field, as other research groups could not reproduce the same results and reached opposite conclusions; thus, this contention has been suspended (reviewed in^52^, ^53^). Importantly, only a portion of the 4‐cell embryos possesses blastomeres that show epigenetic variations,^1^, ^2^ and not all early blastomeres show cell fate bias in lineage‐tracing studies.^49^, ^50^, ^51^ Further, cell fate bias may not be observed among all mouse strains.^54^ Given such heterogeneities among early blastomeres with their great developmental flexibility, it is presumed that a part of early blastomeres—until approximately the 16‐cell stage—tends to promote the differentiation pathway toward one cell lineage, but the differentiation pathway is still reversible and can be canceled. Their differentiation potency is not irreversibly restricted, although they begin to express lineage marker genes and adapt to their positional changes by flexibly changing their fates. Using live imaging of reporter mouse lines, several reports showed that expression of marker genes critical for cell differentiation in the early blastomere not necessarily leads to cell fate determination. It was reported that Nanog, which is an EPI marker gene with a protein product that is indispensable for the maintenance of pluripotency of EPI, starts to be expressed in blastomeres at approximately the 4‐cell stage at random, but its expression levels until the morula stage have no correlation with future cell fates.^55^ Further, it was demonstrated that the morula stage blastomeres expressing Cdx2, which is an important gene for TE differentiation, occasionally cease to express Cdx2 and participate in the ICM and differentiate into both EPI and PrE.^26^

## 5 CELL FATE DETERMINATION OF TROPHOBLAST CELLS

**FIGURE 2:**
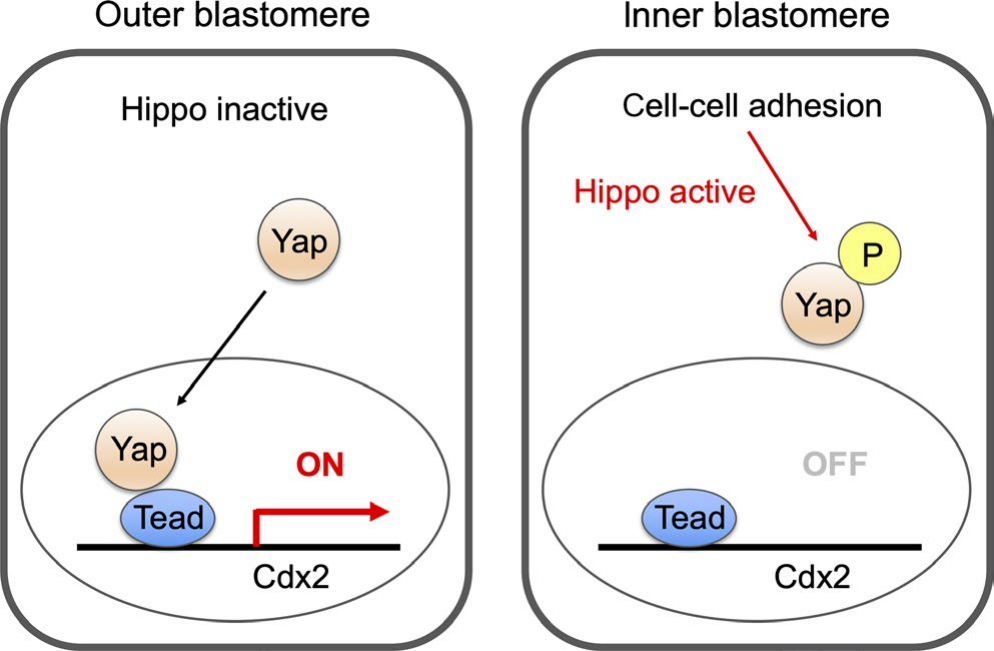
Regulation of Cdx2 expression by cell‐cell contacts via Yap nuclear translocation. In inner blastomeres, Hippo signaling pathway is activated by cell‐cell interactions and prevents the translocation of Yap by phosphorylation. In outer blastomeres, Hippo signaling becomes inactive, and Yap is translocated to the nucleus and forms a complex with Tead4. The Tead4‐Yap complex induces the expression of Cdx2 and other TE genes.

For the cell fate segregation of TE and ICM, two models have been suggested: the positional (inside‐outside) model originally proposed by Tarkowski^4^, ^43^, ^56^ and the polarity model proposed by Johnson.^3^, ^4^, ^56^, ^57^ In the positional model, the cell position within the embryo (inner or outer) determines cell fate. Several studies have revealed that the Hippo signaling pathway plays a central role in mediating positional information of the cells in the early embryo.^58^, ^59^, ^60^, ^61^ Hippo signaling pathway components, the TEAD family transcription factor Tead4 and its coactivator proteins Yap (yes‐associated protein 1) and Taz (transcriptional coactivator with PDZ‐binding motif), are critical for cell fate specification of TE and ICM.^58^, ^59^, ^60^, ^61^ In inner blastomeres, Hippo signaling pathway is activated by cell‐cell interactions and prevents the translocation of Yap. In contrast, in outer blastomeres, Hippo signaling becomes inactive, Yap is translocated to the nucleus and forms a complex with Tead4. Then, the Tead4‐Yap complex induces the expression of caudal type homeobox 2 (Cdx2), which is a transcription factor essential for TE development^60^ (Figure 2), and other genes related to TE development such as Gata3.^62^ These findings at the molecular level seemed to be perfectly consistent with the traditional positional model that cell position plays a critical role in cell fate determination. However, several studies suggested that the mechanism to regulate Hippo signaling pathway is different between early and late stage. Hippo‐active blastomeres (Yap is localized in cytoplasm) were exclusively in inner position at the 32‐cell stage, while considerable number of embryos possessing Hippo‐active blastomeres in the outer position at the 16‐cell stage.^63^, ^64^ It suggests that some factors other than cell‐cell contact govern Hippo activity in the early stage.

**FIGURE 3:**
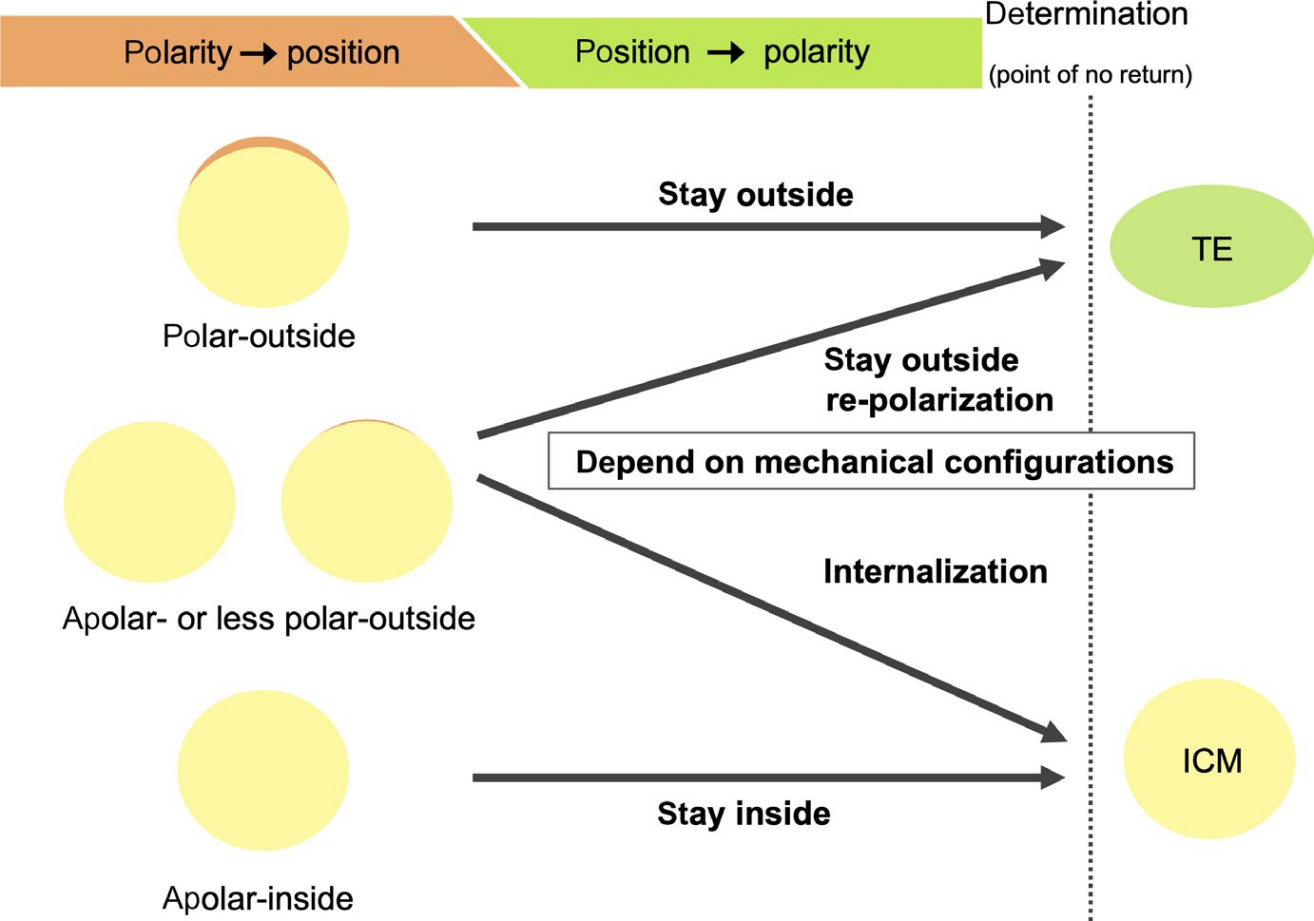
A possible model for roles of cell polarity and positional information in TE/ICM fate specification. Until around the 16‐cell stage, polarity inherited from a parent blastomere governs positional information and direct TE cell determination. Blastomeres at the 8‐cell stage or outer blastomeres at the 16‐cell stage which inherited apical domain (polar‐outside blastomere, the upper cell) inactive Hippo signaling pathway, promote Cdx2 expression, and start TE differentiation. While inner blastomeres without apical domain (apolar‐inside, the lower cell) keep undifferentiated state. Outer apolar or less polar blastomeres (apolar‐ or less polar‐outside, the middle cells) generated by asymmetric division of polarized blastomeres are occasionally internalize probably depending on mechanical configuration of the embryo. Around the 32‐cell stage, positional information becomes dominant and governs polarity. Internalized apolar‐ or less polar‐outside blastomeres activate Hippo signaling pathway, downregulate Cdx2 expression, cease to proceed trophoblast differentiation, and change their fate to ICM.

The polarity model suggests that the differences among the blastomeres in their inheritance of this apical (polarized) surface at cell division, that is, the angle of cell division, determine the developmental fate.^4^ In this model, an asymmetric division of a polarized blastomere (8‐cell stage blastomeres or outer blastomeres at the 16‐cell stage) produces daughter blastomeres that inherit different amounts of apical domains as cell fate determinants that will decide their final fate. Daughter blastomeres that inherit apical domain generate TE, while apolar (not polarized) daughter blastomeres give rise to ICM. Several reports indicate the importance of cell polarity in terms of the Par‐aPKC system in TE and ICM fate determination.^65^, ^66^ Hirate et al revealed a molecular mechanism to transduce polarity information to Hippo signaling pathway.^67^ They demonstrated that the asymmetric localization of angiomotin (Amot) and angiomotin‐like 2 (Amotl2), as governed by cell polarity molecules is critical for the establishment of position‐dependent Hippo signaling.^67^ It suggests the idea that cell polarity—rather than position—dictates Hippo activity in the early stage, which elucidates the interaction between cell polarity and inside‐outside configuration. In particular, Korotkevich et al elegantly showed that the inheritance of an apical domain alone is sufficient to direct the cell fate of the blastomere to TE by transplantation of the apical domain of polarized 8‐cell stage blastomeres to the yet apolar 8‐ and 16‐cell stage blastomeres.^68^ The transplanted apical domain that is monitored by the ezrin signal induced asymmetric division of the apolar blastomere and the daughter blastomere that inherited the apical domain was concluded to progress the TE differentiation process by the expression levels of Cdx2 or the cell envelopment process.^68^ However, it was also reported that apolar blastomere on the outer position could be raised by asymmetric cell division at approximately the 16‐cell stage, and could either be relocated to the inner position and become ICM cells^63^, ^69^ or remain in the outer position and repolarize (acquire apical domains).^68^ This outer apolar blastomere has active Hippo signaling pathway when it is raised by asymmetric cell division, but a part of them remain in the outer position, repolarize, and presumably differentiate to TE.^63^ This fact that outer apolar blastomeres can newly acquire apical domains cannot be explained only by the traditional polarity model, as it is assumed that they recognize their position via cell‐cell contact information and then acquire polarity. From this information, it is speculated that the polarity that is inherited from a parent blastomere first acts as an upstream of the Hippo signaling pathway and directs TE differentiation and that the outer apolar blastomeres are either internalized or remain in the outer position to adjust mechanical configuration of the whole embryo. Finally, the positional information via cell‐cell contact becomes dominant and directs the apolar blastomeres remaining in the outer position to acquire polarity and to change activity of Hippo signaling pathway, and then these blastomeres proceed TE differentiation (Figure 3).

## 6 REVERSIBILITY OF THE TROPHOBLAST DIFFERENTIATION PATHWAY

**FIGURE 4:**
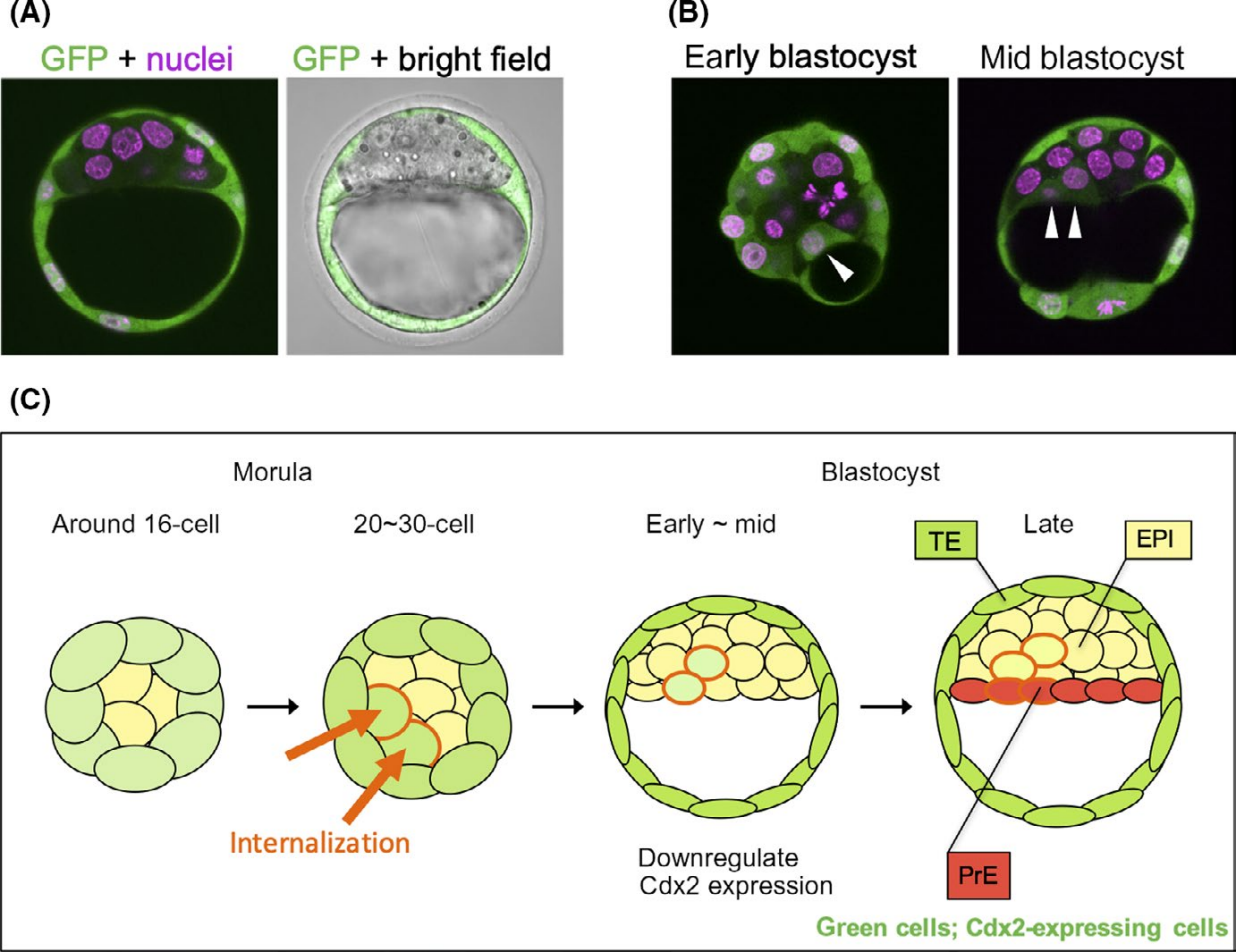
Cdx2 expression in blastomeres presumed to be internalized at the morula‐early blastocyst stage. (A) Typical images of a mid‐blastocyst of a *Cdx2*‐GFP reporter mouse line without inner GFP (*Cdx2*)‐positive cells. GFP fluorescence is exclusively observed in TE layer. (B) Examples of reporter embryos containing inner GFP (*Cdx2*)‐positive cells. *Cdx2* (GFP)‐positive cells in these blastocysts (indicated by white arrowheads) are presumed to be descendants of internalized‐outside blastomeres, that is, originally located in outside and then internalized. (C) A scheme presenting of a summary of presumptive expression patterns of Cdx2 in the internalized‐outside blastomeres. Internalized‐outside blastomeres express Cdx2 when in an outer position, downregulate Cdx2 after internalization, participate in formation of ICM, and contribute to epiblast (EPI) and/or primitive endoderm (PrE).

**FIGURE 5:**
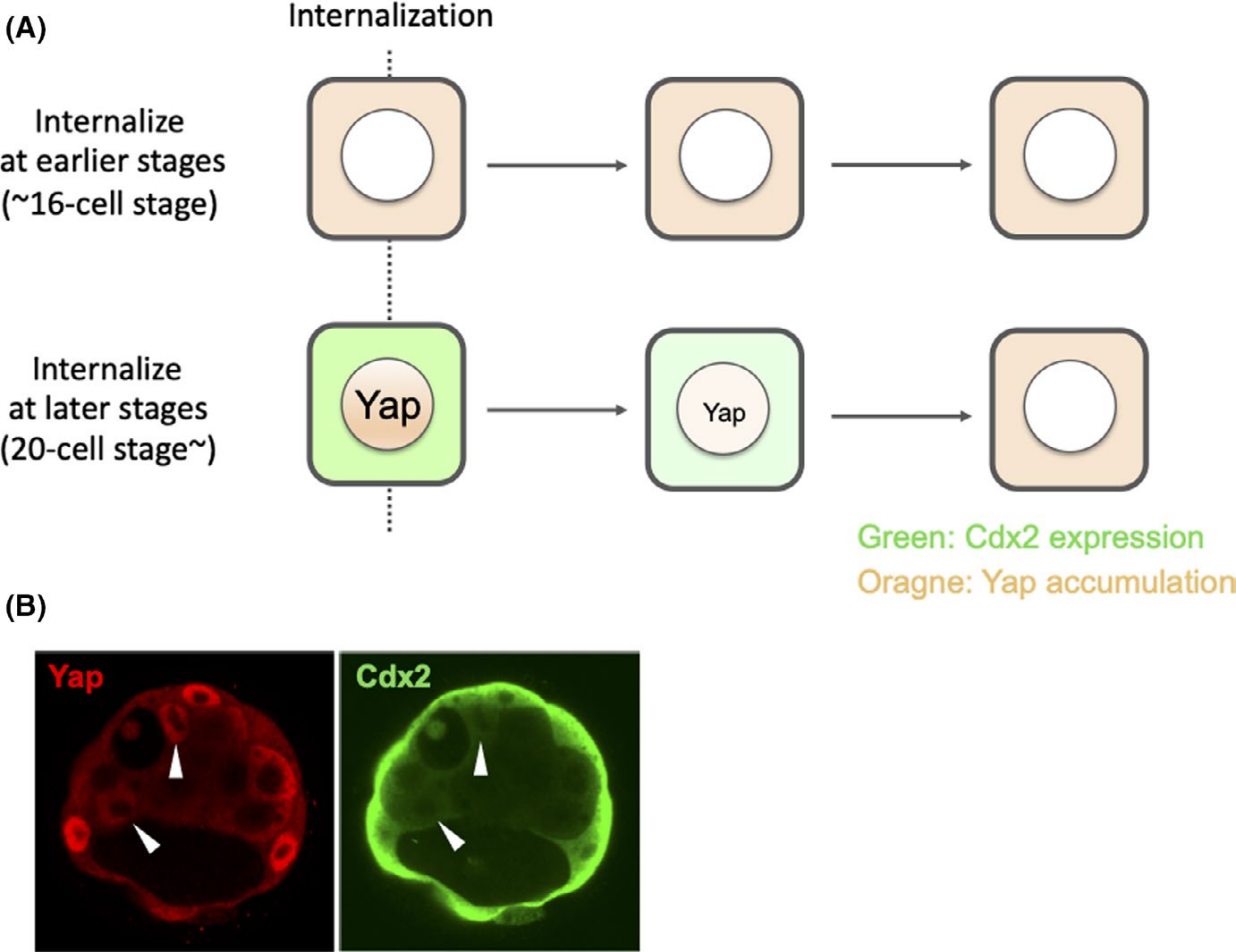
Cdx2 expression and Yap localization in internalized blastomeres. (A) Chronological change of Cdx2 expression and Yap localization in internalized blastomeres in the early and late stage. The upper cell represents blastomeres internalized at the early stage (until around 16‐cell stage) observed by Anani et al. Such a blastomere has active Hippo signaling pathway (cytoplasmic Yap) and presumed to hardly express Cdx2 at the time of internalization. The lower cell represents blastomeres staying outside until the late stage and then are internalized (late‐internalized blastomeres) observed by Toyooka et al. Such a blastomere is presumed to have nuclear Yap and express a considerable amount of Cdx2 at the time of internalization. But after internalization, Yap is translocated to cytoplasm and then Cdx2 in downregulated. (B) Yap and Cdx2 (detected by GFP) localization in the blastomeres in a mid‐blastocyst of *Cdx2*‐GFP reporter mouse. In inner GFP‐positive cells presumed to be descendants of late‐internalized blastomeres (indicated by white arrowheads), Yap is still accumulated in the nuclei of these cells in contrast to other inner blastomeres in which Yap is localized in cytoplasm.

Cdx2 is not just a marker molecule for TE, but also a key transcription factor that is dispensable for early blastocyst formation.^60^, ^70^, ^71^ Artificial expression of Cdx2 in embryonic stem cells induces TE cell differentiation,^72^ which indicates it can operate as a switch to TE differentiation in the preimplantation embryo. Several reports have examined the expression of the Cdx2 protein during TE cell specification by immunostaining.^72^, ^73^, ^74^, ^75^ In these studies, Cdx2 protein was first detected at the 8‐ to 16‐cell stage, and although the staining pattern and intensity in each cell were quite variable among embryos, there was a clear tendency for Cdx2 levels to increase in outer blastomeres and decrease in inner blastomeres.^73^, ^74^ It has been well noted that Cdx2 expression can be detected in some inner blastomeres during the morula to the mid‐blastocyst stages.^73^, ^74^, ^75^ We established the Cdx2‐GFP reporter mice line to visualize and serially observe *Cdx2*‐expressing cells in living embryos (Figure 4A), and found that GFP (Cdx2) ‐positive cells were in the inner side of the early blastocysts of reporter mice^26^ (Figure 4B). From the live‐cell imaging experiments that used preimplantation blastocysts obtained from Cdx2‐GFP reporter mice, we found that such a Cdx2‐positive cell is the descendant of “late‐internalized blastomere” that had initially been in outside positions, and was then internalized at around the 20‐ to 30‐cell stage. The late‐internalized blastomeres, which had expressed Cdx2 when in the outer position, downregulated Cdx2 and behaved as a part of the ICM after relocating to inner position^26^ (Figure 4C). Our observations demonstrate that, although the TE specification pathway is initiated in all the outer cells at approximately the 16‐cell stage, but this process is reversible. A considerable amount of the Yap signal was detected in the nuclei of the late‐internalized blastomeres^26^ (Figure 5A,B), in contrast to the surrounding Cdx2‐negative inner cells in which Yap was excluded from the nuclei, which suggests that the late‐internalized blastomeres were in a transition between outer and inner cell states. Thus, the change in the Yap protein distribution pattern from a nuclear to cytoplasmic pattern in the late‐internalized blastomeres further supports our findings that blastomeres just after internalization gain ICM, but shed TE characteristics.^26^ The ability to reverse a differentiation pathway toward TE could be due to the utilization of a reversible reaction as the switch for TE differentiation, that is, Yap phosphorylation in this case, for transducing information of polarity and cell‐cell interactions from the cell surface to the nucleus. Actually, Nishioka et al demonstrated that blastomeres can change Yap localization responding to positional change within the embryo.^60^ They generated large chimeras by reaggregation of blastomeres from three different 8‐cell stage embryos to force outer blastomeres to occupy an inside position. In these reaggregated embryos, nuclear Yap and Cdx2 were detected only in outside cells, while neither nuclear Yap nor Cdx2 were detected in inside cells of in these reaggregated embryos.^60^ This fact that reaggregated 8‐cell blastomeres, all of which have nuclear Yap, can form cells possessing cytoplasmic Yap indicates that Yap localization can change from the nucleus to cytoplasm. Also, they showed that disruption of E‐cadherin‐mediated cell adhesion by using the E‐cadherin blocking antibody ECCD1 led inside cells to obtain nuclear Yap,^60^ implying that Yap localization can change from cytoplasm to the nucleus. These results suggest that Yap translocation between the nucleus and cytoplasm is reversible process in the blastomeres of the preimplantation embryo.

Due to the persistence of GFP fluorescence in the late‐internalized blastomeres at the late blastocyst stage, we could trace the subsequent fate of the these blastomeres. We examined the cell fates of the late‐internalized blastomeres using markers for presumptive EPI and PrE. We found that the late‐internalized blastomeres differentiated into both presumptive EPI and PrE precursors, which indicates that these blastomeres could differentiate into both the EPI and PrE.^26^ Previous live‐imaging studies that visualize internalization of blastomeres in preimplantation embryos showed that the inner blastomeres at the 16‐cell stage had been produced not only by asymmetric division, but also by the internalization of daughters of the 8‐cell blastomeres that were initially located at the outer position.^63^, ^76^, ^77^, ^78^ It has also been shown that the internalization process occurs after the 16‐cell stage,^76^, ^79^ which suggests that internalization of blastomeres is essential for the establishment of the outer‐inner configuration. However, it remains unclear whether the internalizing blastomeres are different from other blastomeres keeping outer position at the time of internalization. A study has suggested that differences in Notch activity among blastomeres cause the differences in their relative position within an embryo.^80^ If this is the case, outside blastomeres with weaker Notch activity may tend to be internalized. The driving force for internalization has also not yet been fully elucidated, but it was suggested that apolar‐ outer blastomeres have higher surface tension and higher contractility, which drive blastomeres into the inner position of the embryo.^63^, ^81^

Anani et al found that a population of the apolar blastomeres that are located at the outer positions until the 16‐cell embryo stage (apolar‐outer blastomeres) were eventually internalized,^63^ which suggests that the outer‐inner configuration is regulated by polarity of the blastomeres at least until the 16‐cell stage. They also showed that the Hippo pathway is already active in such apolar‐outer blastomeres while on the surface of embryos (Figure 5A) and that such apolar‐outer blastomeres that demonstrate high Hippo activity were not observed in the outer positions after the late morula stage.^63^ On the other hand, we found that a considerable amount of Yap proteins persisted to the nuclei in the descendant cells of late‐internalized blastomeres (Figure 5A,B), which are presumed to be internalized at the late morula stage and which implicates that the Hippo pathway was inactivated in these blastomeres before internalization, but activated after internalization.^26^ Taken together, it is supposed that the Hippo pathway is first active in apolar‐ or less apolar‐outer blastomeres at around the 16‐cell stage as Anani et al reported, and if they are not internalized and stay outside until the later (around 20‐30 cell) stage, they repolarize, the Hippo pathway becomes inactive, and Cdx2 expression is upregulated in such blastomeres as reported by us.^26^, ^63^ Overall, these observations are coincident with the idea mentioned above that first cell polarity governs the activity of the Hippo pathway, then cell‐cell interactions increasingly govern the activity of the Hippo pathway, and finally determine the cell fate at the late stage.

**FIGURE 6:**
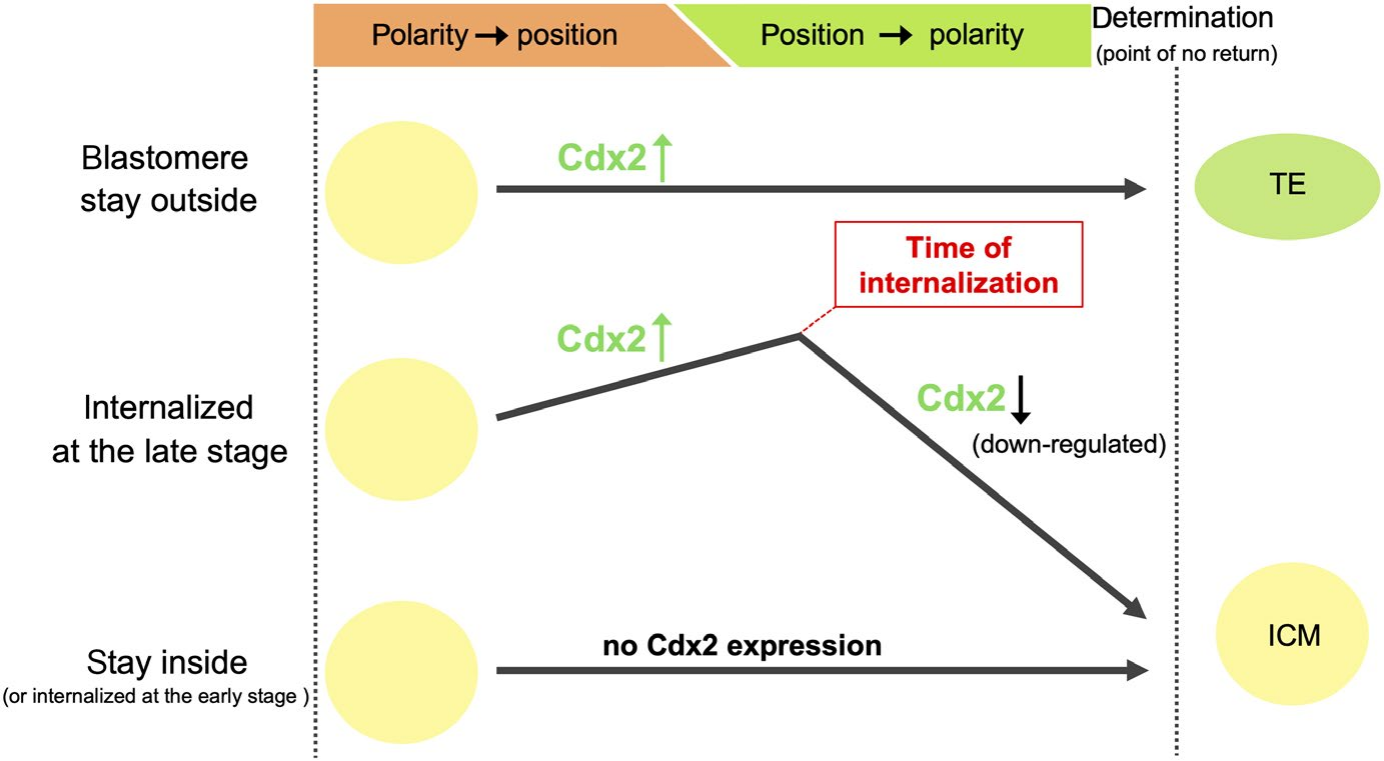
Summary of Cdx2 expression patterns in blastomeres during the mouse preimplantation embryo. Blastomeres which keep outside position throughout preimplantation period upregulate Cdx2 expression and differentiate into TE. Blastomeres which stay inside throughout preimplantation period (or blastomeres internalized at the early stage) do not (or hardly) express Cdx2 and finally give rise to ICM. Around the 32‐cell stage, positional information becomes dominant and governs polarity. Blastomeres internalized at the late stage express a considerable amount of Cdx2, but downregulate Cdx2 after internalization, cease to proceed trophoblast differentiation, and change their fate to ICM. All blastomeres until around the 32‐cell stage are in “tentative state” in which they possess plasticity and change their fate even if they express Cdx2. Irreversible cell fate determination to TE/ICM occurs at around the 32‐cell stage.

Thus we propose a “tentative state” in which a blastomere that is progressing in the differentiation pathway begins to change morphology or express lineage marker genes, which does not irreversibly determine their cell fate. Given TE cell specification, it is presumed that first cell polarity promotes differentiation pathway toward the TE cell fate in outer polarized blastomeres, and blastomeres that possess apical domain abundantly flatten their cell shape early and dominantly occupied the surface of the embryo. A part of the outer blastomeres that include a part of the apolar blastomeres covered by early‐flattened blastomeres, lose their outer surface, and are consequently internalized. Internalized blastomeres until around the 16‐cell stage (internalized at the early stage) hardly express Cdx2 and are presumed to become ICM cells. After around the 32‐cell stage, positional information becomes dominant and governs polarity. Internalized blastomeres, which stay outside after around 20‐cell stage and then are internalized (internalized at the late stage), are presumed to express a considerable amount of Cdx2, but downregulate Cdx2 expression after internalization, cease to proceed trophoblast differentiation, and change their fate to ICM (Figure 6). We suppose that a blastomere that begins differentiation to the TE cell fate early may predominantly occupy the surface of the embryo, but all blastomeres until the 32‐cell stage are in the tentative state and possess differentiation potency for both TE and ICM, even though genes critical for TE differentiation—such as Cdx2—are expressed

## 7 HUMAN TE DEVELOPMENT AND TROPHOBLAST STEM CELLS

The mouse preimplantation embryo contains three cell lineages: pluripotent epiblast cells that give rise to embryonic tissues (EPI cells), primitive endoderm cells that mainly form embryonic membranes (PrE cells), and trophoblast cells that form the TE layer (TE cells). The preimplantation development of both human and mouse blastocysts is nearly identical, but some differences do exist; for instance, the timing of the zygotic genome activation (ZGA), at which the activation of embryonic transcription begins, is different between mice and humans. The major wave of ZGA occurs at the late 2‐cell stage in the mouse, while at the 8‐cell stage in human embryos.^82^In addition, some transcription factors critical for TE and ICM differentiation in the mouse embryo are also expressed in the human embryo, but not prior to the blastocyst stage. Lineage‐restricted expression of marker genes, such as CDX2, OCT4, NANOG, and GATA6, does not occur until the late blastocyst stage in the human embryo.^83^ Consistent with this late restriction of the expression of marker molecules, isolated TE and ICM from human early blastocysts were found to regenerate both (TE and ICM) cell types,^84^ while inside cells from the late morula can regenerate TE, but outer cells from the late morula or early blastocyst are already restricted to TE in mice.^44^

It is generally known that stem cell lines, the embryonic stem cells (ESCs), trophoblast stem cells (TSCs), and extraembryonic endoderm stem cells (XENCs) can be established from these three cell types in mouse preimplantation embryos and have distinct features and are based on different signaling pathways. ESCs are a pluripotent immortal cell line that is derived by the culture of the ICM cells—originally in the media that contain the cytokine leukemia inhibitory factor (LIF). They express pluripotent markers—such as Oct4 or Nanog—and can contribute to all tissues in the chimeric mouse. The signaling pathway in which LIF inhibits differentiation and promotes the self‐renewal of ESC is well studied. Further, LIF signaling is transduced via the heterodimer of LIF receptor (LIFR) and the common subunit gp130.^85^ Binding of LIF to the LIFR‐gp130 heterodimer activates the transcription factor STAT3 via the JAK‐STAT signaling pathway, and activated STAT3 translocates to the nucleus and is involved in the gene expression that is required for the maintenance of pluripotency.^86^, ^87^ Meanwhile, the LIF‐LIFR/gp130 signaling pathway also activates the Erk signaling pathway. Stimulation of the Erk pathway through both the LIF‐LIFR/gp130 and FGF4‐FGFR2 pathways is known to be essential for differentiation of ESC.^88^, ^89^, ^90^ Austin Smith and colleagues showed that inhibition of the Erk signal with small molecules is sufficient to maintain the pluripotent state of ESC; in addition, the medium that was developed by this research group contains small molecules that block the Erk pathway and activate Wnt pathway by suppression of the activity of the glycogen synthase kinase‐3 (Gsk3), and has been widely used for the maintenance of ESC since a decade ago.^91^, ^92^ While TSC is derived from both the pre‐ and post‐implantation embryos by culturing in the presence of the fibroblast growth factor 4 (FGF4) heparin and using the fibroblast cells as the feeder cells or their conditioned medium.^93^ TSC can self‐renew, express marker genes of early diploid trophoblasts—such as Cdx2 or Eomes—and can differentiate toward the giant cell when FGF4 is removed from the medium. These cells can exclusively contribute to the trophoblast cell lineage in the placenta and not to the fetus in the chimera that is made by injection into the blastocyst.^93^ XENC can be derived from the blastocyst in the presence of FGF4, heparin, and feeder cells in the same conditions as the TSC derivation.^94^ XENC can self‐renew, express marker genes for the extraembryonic endoderm—such as Gata6 or Gata4—and demonstrate exclusive contribution to the extraembryonic endoderm lineages in the chimera. Unlike TSC, the maintenance of established XENC requires the feeder cells or its conditioned medium, but not FGF4 and heparin, which suggests that they do not depend on the FGF4 signal for their self‐renewal.^94^ The establishment of such stem cell lines revealed the factors and signaling pathways that these cells require in vivo.

Attempts to establish human TSC using the human trophoblast have been performed by several researches without success. In recent years, Okae et al reported the derivation of human TSC from human blastocyst and early placenta.^95^ They initially analyzed transcriptome of cytotrophoblast (CT) cells, which were thought to comprise an undifferentiated cell population in the early placenta, and determined signaling pathways that were required to maintain an undifferentiated state. Based on this analysis, they succeeded in determining culture conditions in which TSC could be derived from primary CT cells of the early placenta and maintained for at least 5 months. CT cell‐derived human TSC could differentiate into in vivo derivatives of CT cells, that is, extravillous trophoblast (EVT)‐like cells, and into syncytiotrophoblast (ST)‐like cells in vitro. Using the same culture conditions, the establishment of TSC from the human preimplantation blastocyst was finally successful. Their culture medium for TSC contains GSK3 inhibitors (a Wnt activator), epithelial growth factors (EGF), transforming growth factor‐beta (TGF‐β) inhibitors, histone deacetylase (HDAC) inhibitors, and Rho‐associated protein kinase (ROCK) inhibitors, while mouse TSC requires FGF4 and heparin.^93^, ^95^ Overall, given that human TSC could not be derived in mouse TSC conditions,^96^ it is suggested that the factors and signaling pathways that are essential for human and mouse TSCs are different despite their similar morphology. Interestingly, this contrasts with the fact that both human ESC and its mouse analog, termed mouse epiblast stem cell (EpiSC), which is derived from the epiblast of the post‐implantation embryo, similarly require basic fibroblast growth factors (bFGF) and activin.^97^, ^98^, ^99^, ^100^ However, human TSC poorly expresses CDX2, EOMES, and SOX2,^95^ which are genes that encode transcription factors thought to be required for mouse TSC self‐renewal.^101^ Further examination is needed to characterize human TSC and to research the function of these transcription factors for self‐renewal of TSC. Since the establishment of human TSC requires human blastocysts or early placenta—both of which are rarely available—TSC‐like cells derived from human ESC or human iPSC may be easier to use for research at the present time.^102^ In addition, some research groups reported generation of blastocyst‐like structure, termed blastoids or blastocyst‐like cysts (iBLCs) in vitro, by combining mouse TSC and other stem cells derived from mouse preimplantation embryos or solely from mouse EpiSC, respectively.^103^, ^104^, ^105^ These structures morphologically and transcriptionally resembled blastocysts although they could not generate live‐born mice when they were transferred to the uterus,^103^, ^104^, ^105^ expected to provide a useful system for research of mechanisms of TE development and implantation.

## 8 CONCLUSIONS

For TE specification, there are two traditional models—the positional model and the polarity model. These models are not exclusive and instead can be complementary, and their hierarchical relationship is being resolved. In this article, we proposed a “tentative state,” in which a blastomere proceeds to undergo TE cell differentiation (in terms of changes in morphology and expression of marker genes) but is not irreversibly restricted and can change its cell fate to ICM. All blastomeres until approximately the 16‐cell stage are supposed to be in the “tentative state,” even if they show heterogeneity in their epigenetic modifications or gene expression patterns. Considering TE specification with this concept, first cell polarity initiates differentiation toward the TE cell fate, and later, when positional information becomes dominant, all outer cells start to express Cdx2. A part of outer cells is internalized, downregulates Cdx2, ceases to proceed along the TE differentiation pathway, and participates in ICM formation. Further, we introduced human TSC, which is a material that is expected to be useful for both basic and medical research. Observation of mouse embryos and studies that use mouse and human TSC or TSC‐like cells will allow a further understanding on TE development before and after implantation.

## DISCLOSURES

*Conflict of interest*: The author declares no conflict of interest. *Human rights statements and informed consent*: This article does not contain any studies with human participants. *Animal study*: All the animal experiments in this article were approved by the Ethical Committee for Animal Experiment of NIBB.
